# Parafoveal cone function in choroideremia assessed with adaptive optics optoretinography

**DOI:** 10.1038/s41598-024-58059-x

**Published:** 2024-04-09

**Authors:** Peiluo Xu, Robert F. Cooper, Yu You Jiang, Jessica I. W. Morgan

**Affiliations:** 1 Department of Bioengineering, University of Pennsylvania, Philadelphia, PA 19104 USA; 2 Scheie Eye Institute, University of Pennsylvania, Philadelphia, PA 19104 USA; 3 Department of Ophthalmology, Joint Department of Biomedical Engineering, Medical College of Wisconsin, Marquette University and Medical College of Wisconsin, Milwaukee, WI 53233 USA; 4 Center for Advanced Retinal and Ocular Therapeutics, University of Pennsylvania, Philadelphia, PA 19104 USA

**Keywords:** Retina, Hereditary eye disease

## Abstract

Choroideremia (CHM) is an X-linked retinal degeneration leading to loss of the photoreceptors, retinal pigment epithelium (RPE), and choroid. Adaptive optics optoretinography is an emerging technique for noninvasive, objective assessment of photoreceptor function. Here, we investigate parafoveal cone function in CHM using adaptive optics optoretinography and compare with cone structure and clinical assessments of vision. Parafoveal cone mosaics of 10 CHM and four normal-sighted participants were imaged with an adaptive optics scanning light ophthalmoscope. While acquiring video sequences, a 2 s 550Δ10 nm, 450 nW/deg^2^ stimulus was presented. Videos were registered and the intensity of each cone in each frame was extracted, normalized, standardized, and aggregated to generate the population optoretinogram (ORG) over time. A gamma-pdf was fit to the ORG and the peak was extracted as ORG amplitude. CHM ORG amplitudes were compared to normal and were correlated with bound cone density, ellipsoid zone to RPE/Bruch’s membrane (EZ-to-RPE/BrM) distance, and foveal sensitivity using Pearson correlation analysis. ORG amplitude was significantly reduced in CHM compared to normal (0.22 ± 0.15 vs. 1.34 ± 0.31). In addition, CHM ORG amplitude was positively correlated with cone density, EZ-to-RPE/BrM distance, and foveal sensitivity. Our results demonstrate promise for using ORG as a biomarker of photoreceptor function.

## Introduction

Choroideremia (CHM) is an X-linked inherited chorioretinal dystrophy caused by mutation or deletion of the CHM gene, leading to the absence of Rab-escort protein 1 (REP-1). REP-1 is a ubiquitously expressed protein involved in cellular processes as a regulator of signal transduction, protein trafficking, and endocytosis^1,2^. REP-1 is a key mediator of vesicular trafficking, thus its deficiency leads to progressive degeneration of photoreceptors, retinal pigment epithelium (RPE) and choroid^3^.

Fundus imaging in CHM consistently shows a centrally maintained retina and RPE surrounded by peripheral areas of atrophic, dyspigmented retina^4^. Cross-sectional imaging of the retina using optical coherence tomography (OCT) has revealed preserved or thickened but otherwise normally laminated central retina in early stage of the disease, followed by progressive thinning of the central retina in late-stage disease^5,6^. Previous OCT studies of CHM have also shown that the interdigitation layer of the photoreceptors with the apical RPE is disrupted within the centrally maintained retina^7^. Adaptive optics scanning light ophthalmoscopy (AOSLO), an imaging technique that enables high resolution visualization of cellular-scale retinal features^8^, has revealed distinct foveal phenotypes in the cone mosaic including patches of hyper- and hypo-reflective cones, increased cone inner segment diameters, and reduced cone photoreceptor density^9–12^.

Patients with CHM often experience nyctalopia in early childhood, followed by peripheral visual field loss, and ultimately legal blindness around the fifth decade^13^. Best-corrected visual acuity remains relatively stable in affected individuals at young ages but rapidly declines once the disease has progressed into the foveal region at approximately 50 years of age^14–16^. Despite its relatively low sensitivity for assessing disease progression in CHM, best-corrected visual acuity remains a highly utilized clinical procedure for measuring vision and has been the primary outcome measurement for gene therapy clinical trials^17–20^. Microperimetry is also widely used to assess retinal sensitivity at localized regions of the fundus in CHM^21^. In cases where the atrophic border is at least 2.5 mm away from the fovea, foveal sensitivity remains near normal in CHM^22^. Retinal sensitivities close to the atrophic border however, are reduced and locations beyond the atrophic border many times reveal complete scotomas to the testing stimuli^21^. Though retinal sensitivity at different locations of the visual field provides a more complete understanding of visual function than acuity in a CHM patient, microperimetry has shown reduced repeatability at locations near the border of degeneration^21,22^, perhaps because the accuracy with which the testing stimuli can be repeatedly delivered to the same retinal area is less than the transitional area between functional and degenerated retina in this disease. Indeed, adaptive optics (AO) guided microperimetry has revealed steep transitions in retinal sensitivities measured at the atrophic border in correlation with structural images of the remaining photoreceptor mosaic^23^.

Both visual acuity and microperimetry are subjective measures of vision, meaning that responses from the patient are required throughout the procedures. Objective, surrogate measures of visual function that do not require patient input such as electroretinography (ERG), which records the electrical signal arising from the retina in response to photic stimulation^24,25^, are desirable. ERG procedures in CHM affected individuals however yield undetectable responses in many cases^26^, presumably because the ERG integrates signals over large retinal areas and the centrally retained retinal area is relatively small in comparison to the wide-spread peripheral atrophy observed in CHM^21,22,27^. Multifocal ERG (mfERG) provides better spatial resolution, however, to our knowledge, studies of CHM have only reported mfERG results in one carrier of the disease, but not affected individuals^28^.

The emerging field of AO optoretinography may provide a localized, objective measure of photoreceptor function that fills the need for assessing CHM and other retinal diseases. Briefly, optoretinography procedures record an optical signal arising from the retina in response to a visual stimulus. When combined with AO, these measurements can be confined to individual photoreceptors^29^ or aggregated across the local cone mosaic^30,31^ in a given patch of retina. To date, investigators have shown the population optoretinogram (ORG) is repeatable both within and across sessions^31^ and the action spectrum of the ORG is well matched to the human photopic luminosity function^30^. At the level of individual cells, ORG measurements have shown increasing ORG responses with increasing stimuli irradiance and have enabled classification of cone types in the trichromatic mosaic^29,32–34^. Studies of optoretinography largely have been confined to proof of concept studies in normal sighted individuals^29–39^. To our knowledge, there has only been one report of optoretinography applied to retinal disease^40^. In that study, investigators found ORG measurements of cones were reduced in the transition zones of three patients with retinitis pigmentosa and decreased with increasing disease severity^40^. It remains unknown how ORG measurements are impacted in CHM.

In this study, we measured the population ORG of cones in parafoveal retinas in CHM compared to normal-sighted participants using AO optoretinography. We further correlated the ORG from CHM participants with cone densities and foveal sensitivities. In doing so, we provide further knowledge on photoreceptor function in CHM and develop ORG as an objective biomarker for photoreceptor function in retinal disease.

## Methods

### Subjects

This study followed the Declaration of Helsinki and was approved by the institutional review board at the University of Pennsylvania. Ten genetically confirmed CHM (ages: 20–49 years; all males) and four normal-sighted subjects (ages: 30–57 years; three males, one female) participated in the study. Genetic mutations for all participating CHM patients are reported in Wynne et al.^10^ All participants provided informed consent after learning the nature and possible consequences of the study. Only one eye per participant was included in the study. Prior to imaging, each study eye was dilated with phenylephrine hydrochloride (2.5%) and tropicamide (1%). Visual acuity for CHM participants was measured as was foveal sensitivity to a white 200 ms, Goldman III size circular stimuli using the Nidek MP-1 Microperimeter (Nidek, San Jose, CA, USA).

### AOSLO image sequence acquisition

Confocal and nonconfocal split detection and dark-field image sequences were simultaneously collected using a previously described custom-built, multimodal AOSLO^41,42^. The cone mosaic was imaged 0.5–1° from the preferred retinal locus over a 1° × 1° field of view using a 795 nm superluminescent diode imaging beam. A superluminescent diode centered at 840 nm (Superlum, Cork, Ireland) was used for wavefront sensing and a 97-actuator deformable mirror (Alpao SAS, Montbonnot-Saint-Martin, France) was used to correct the optical aberrations. The visible stimulus was delivered to the retina over the full imaging location using a SuperK EXTREME super-continuum laser and VARIA tunable filter centered at 550 nm with a bandwidth of 10 nm (NKT Photonics, Birkerød, Denmark).

Each imaging session consisted of 1–6 stimulus trials, and each trial included 1–13 image sequence acquisitions depending on subject availability. This resulted in a minimum of 6 stimulated acquisitions for each CHM participant. Details of our dataset are provided in Supplementary Table 1. At the beginning of each trial, the subject’s study eye was dark adapted for 2 min. During each acquisition, a two-second, 450 nW/deg^2^, 550Δ10 nm stimulus was presented to the subject four seconds after the recording started, and imaging continued for at least 4 s post-stimulus. Non-stimulated control trials with the same imaging conditions but without a visible stimulus were also collected in all normal-sighted participants and 8 of the 10 CHM participants.

### Image sequence processing

Non-confocal split-detection and dark-field image sequences were generated by subtracting the two semi-annular PMT collections and dividing by their sum or summing the two collections, as previously described^42^. The confocal, non-confocal split-detection, and dark-field AOSLO video sequences then were desinusoided using an image from a static Ronchi ruling and registered using a strip-based technique to alleviate intra-frame eye motion using a previously described custom algorithm^43^ We used the split-detection image sequences for calculating transformation parameters, and applied the same transforms to the simultaneously collected confocal and dark-field image sequences.

Strip-based registration software requires the identification of a reference frame to which every other frame in the sequence is compared. A custom Matlab script was used to automatically select every other frame during the stimulus window as a reference frame and the registrations were batch processed. The registration with the highest number of frames registered was automatically selected and this registration was manually evaluated to ensure the registration was sufficient and accurate. In the event that the registration was deemed insufficient or inaccurate, the registration with the next highest number of registered frames was selected, and the manual evaluation process was repeated. This process was repeated until an acceptable video was found. At least 60% of the frames acquired during the 2 s stimulus window were required to be included in the registration for the acquisition to continue to the next step of analysis. The automated reference frame selection with manual verification registration process resulted in 90% of acquisitions in the study meeting this criterion. We note that this process did not change the final output of the registered video sequences, but did reduce the amount of manual operator labor required to identify an acceptable reference frame for registration.

Following selection of the strip registered image sequence, a frame-wide affine registration was performed on each frame to remove residual torsion. Finally, image sequences were cropped to a common area for all the images collected within the imaging session.

### ORG signal extraction

**Figure 1:**
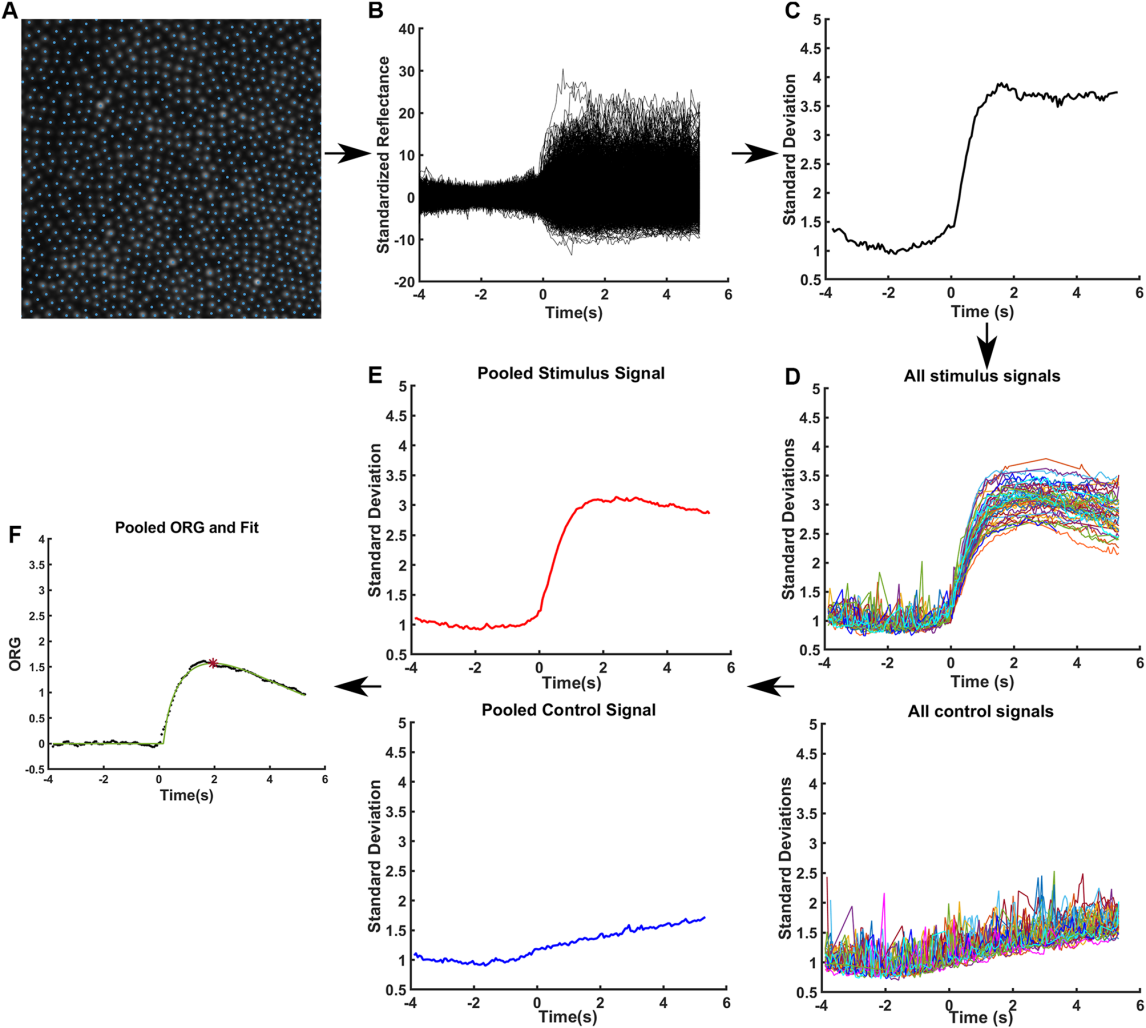
AOSLO confocal image sequence processing and ORG signal extraction. (**A**) An exemplar confocal image of the common area averaged over all the video acquisitions from a single session for a normal-sighted participant. Cone centers were identified (blue dots). (**B**) Normalized and standardized intensity from every identified cone from one image sequence. (**C**) Standardized intensities were aggregated over all the identified cones by calculating the standard deviation of all traces shown in (**B**) at every time point. (**D**) Signals from multiple acquisitions acquired during a single session where each line comes from a unique acquisition processed in the same manner as (**C**). (**E**) The pooled standard deviation at every time point for both stimulated acquisitions (red) and control acquisitions (blue). (**F**) The control curve is subtracted from the stimulated curve, and the result is referred to as the ORG (black). A gamma-pdf is fit (green) to the ORG signal (black) and the peak of this fit is taken as the ORG amplitude (red asterisk).

The ORG was extracted using a previously described method, which we summarize here (Fig. 1)^30^. An average image of the common area was generated for the confocal, split detection and dark-field imaging modalities for each registered video sequence. On each image triplet, we identified the cones in a semi-automatic fashion using MOSAIC (Translational Imaging Innovations, Hickory, NC, USA). With this tool, we applied a deep neural network^44^ on the confocal image to automatically identify most of the cones. Manual adjustments to these automatic identifications mainly based on the split-detection image with the confocal image available as a secondary reference were performed to correct for any misidentified cones (Fig. 1A). In addition to extracting the intensity traces for each cone over the image sequences (see below), we measured the bound cone density of the mosaic for each participant by counting the number of cones within the bound Voronoi area of a 55 µm per side square sub-region of the same image and dividing by the bound Voronoi area.

For ORG measurements, we excluded cones underlying capillaries by creating a binary mask highlighting the vasculature features present^45^. Cell signals (intensity vs. time) were extracted by averaging over a 3 × 3 pixel region centered at each cone coordinate for each frame. To account for frame-wise changes in intensity that could occur through various factors such as tear film disruptions and AO correction, each cone’s intensity was normalized by dividing its intensity by the mean intensity of all analyzed cones in that frame. The cone normalized intensities then were standardized by subtracting their pre-stimulus mean and dividing by their prestimulus standard deviation (Fig. 1B)^30^. We aggregated the signals across individual cones by calculating the standard deviation across all cones’ standardized intensity signals at each time point to obtain a population response (Fig. 1C). We repeated this analysis for every acquisition, and combined the signals from all acquisitions from the same subject using the pooled standard deviation defined as the square root of the mean squared cone intensities (Fig. 1D). The same analysis was performed on control trials (Fig. 1E) if available. We then subtracted the control signal from the stimulated signal to isolate the signal arising from the visible stimulus. We refer to this subtracted trace as the ORG. Finally, a gamma probability density function (gamma-pdf) was fit to the ORG to produce a smooth trace without any noise spikes and the peak of the fit was taken as the ORG amplitude (Fig. 1F).

We did not collect control trials in 2 out of 10 CHM participants. For these two participants, we used the mean of the control trials from the other 8 CHM participants in the study as the control signal.

### Optical coherence tomography

Cross-sectional images of the retina were acquired using a Heidelberg Spectralis Optical Coherence Tomography (OCT) device. OCT images were aligned to the AO montage and the retinal location where the ORG was measured was identified. We used a custom MATLAB script to extract reflectivity as a function of depth centered on the retinal location where the ORG was measured. Reflectivity was averaged over a 14-pixel lateral region (approximately 55 µm) at each depth. The reflectivity peaks corresponding to the ellipsoid zone and the retinal pigment epithelium (RPE)/Bruch’s membrane layers were identified and the ellipsoid zone to RPE/Bruch’s membrane (EZ-to-RPE/BrM) distance was extracted.

### Statistical tests

All statistical analysis was done using MATLAB (MathWorks, Natick, MA, USA). ORG amplitudes were compared between CHM and normal-sighted participants using unbalanced one-way ANOVA and Bonferroni post hoc test, with p < 0.05 considered significant. Linear regression with Pearson’s correlation coefficient (PCC) was used to assess the correlation between bound cone density, EZ-to-BrM distance, and foveal sensitivity against ORG amplitude in CHM participants.

## Results

**Figure 2:**
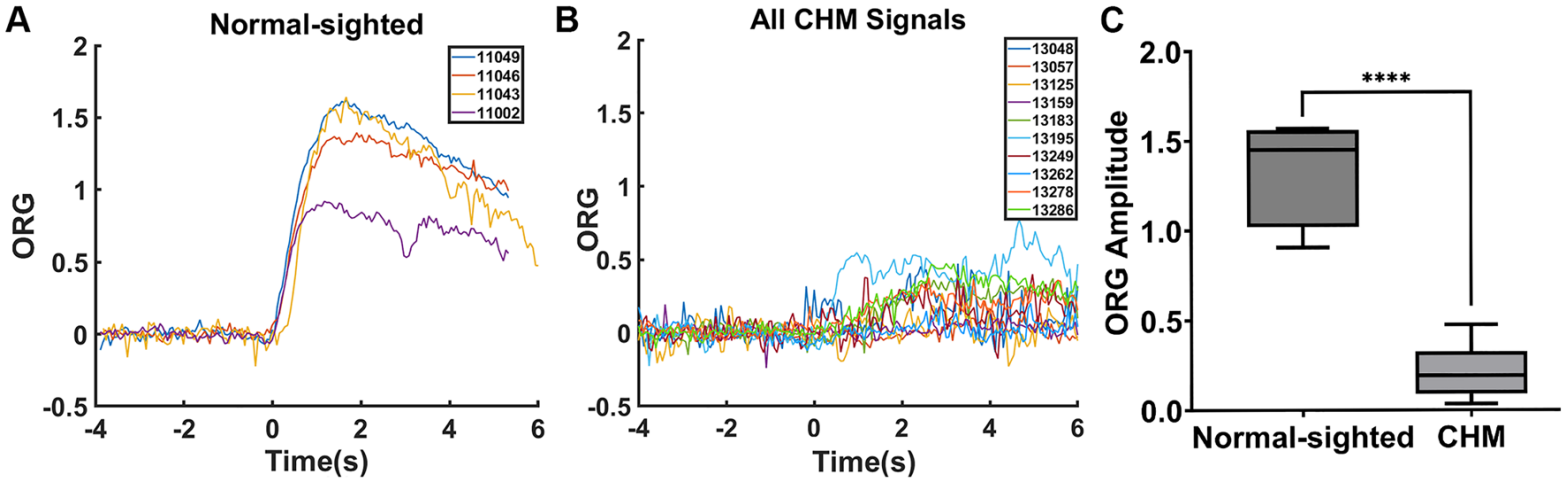
ORGs from (**A**) normal-sighted participants and (**B**) CHM participants. Box and whisker plots (**C**) illustrating ORG amplitudes from CHM participants are significantly reduced in comparison to ORG amplitudes from normal-sighted participants, p < 0.001. The line inside the box represents the median ORG amplitude for each group. Lower and upper box boundaries represent the 25th and 75th percentiles respectively, and the lower and upper error lines show lowest and highest ORG amplitude respectively.

Age, visual acuity, ORG amplitude, foveal sensitivity, EZ-to-BrM distance, and cone density is reported in Table 1. ORGs were successfully recorded as described for all study participants. The ORGs of CHM study participants were different from normal-sighted participants in that the change in intensity in CHM cones following the presentation of the visible stimulus was less apparent compared to that in normal-sighted participants. In all normal-sighted participants, ORGs exhibited a steep increase upon stimulus onset and reached a peak at approximately the same time as the stimulus was turned off (2 s) (Fig. 2A, Supplemental Fig. 1). In all CHM participants, however, ORGs appeared compromised with more gradual increases throughout the stimulus duration, lower peak amplitudes, and in some cases a delay in the peak amplitude beyond the 2 s stimulus (Fig. 2B, Supplemental Fig. 1). ORG amplitudes extracted from gamma-pdf fits were significantly lower in CHM participants (mean ± standard deviation, 0.22 ± 0.15, arbitrary units, range 0.04–0.48) compared to normal-sighted participants (1.34 ± 0.31, arbitrary units, range 0.91–1.57) with p < 0.001 (Fig. 2C). For three CHM participants, the ORG appeared essentially flat, with amplitudes lower than 0.1 (Table 1).

**Table 1:** Age, visual acuity, ORG amplitude, foveal sensitivity, Distance from ellipsoid zone to RPE/Bruch’s membrane (EZ-to-RPE/BrM), and cone density for CHM (top) and normal-sighted (bottom) participants.

Participant	Age	Visual acuity	ORG amplitude	Foveal sensitivity (dB)	EZ-to-RPE/BrM (µm)	Cone density ( cells/mm^2^)
13048	27	20/20–2	0.2685	10	43	48,490
13057	36	20/32 + 2	0.1010	10	27	31,210
13125	34	20/20–2	0.0350	10	39	59,970
13159	45	20/25	0.0645	10	31	31,430
13183	43	20/20	0.3162	20	46	71,050
13195	38	20/20	0.4825	18	46	68,240
13249	20	20/20	0.1548	12	39	62,100
13262	38	20/30–2	0.0773	8	27	27,050
13278	49	20/20–2	0.2668	20	31	51,720
13286	29	20/20–3	0.3811	2*	58	87,540
11049	30	–	1.5691	–	50	80,507
11046	57	–	1.3649	–	54	76,870
11043	34	–	1.5348	–	54	86,253
11002^†^	37	–	0.9064	–	58	65,388

*The retinal sensitivity measured across the macular region using the Nidek microperimeter in patient 13,286 appeared atypical for CHM. Retinal sensitivities measured with the MAIA microperimeter on the same day showed the expected pattern over the central island. Foveal sensitivity measured with the MAIA for this patient was 23 dB. Patient fatigue is the likely cause of the (believed inaccurate) Nidek result.

^†^Female normal-sighted participant.

**Figure 3:**
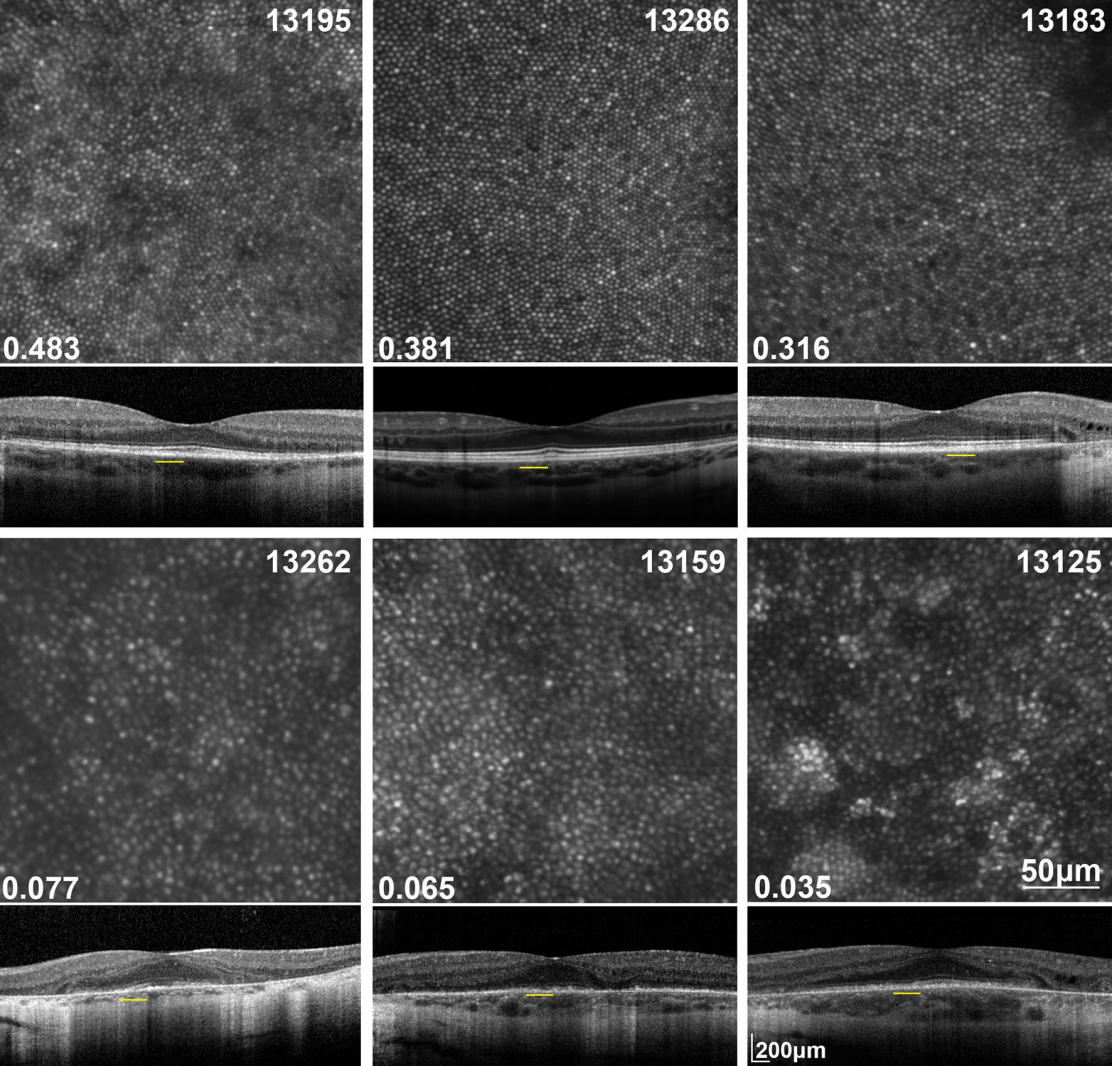
AO images of the cone mosaic and corresponding OCT images where ORGs were measured from the CHM participants with the three highest ORG amplitudes (top) and the three lowest ORG amplitudes (bottom). The number in the lower left corner of each AO image panel indicates the ORG amplitude for that particular participant. Yellow line under the OCT indicates the retinal location of the AO image.

Structural images of the photoreceptor mosaic qualitatively appeared to correlate with ORG findings. CHM participants with higher ORG amplitudes exhibited a more contiguous parafoveal cone mosaic with cone intensities that closely resembled images of normal parafoveal cones. On the other hand, cone mosaics from CHM participants with lower ORG amplitudes showed relatively larger spacing between cones and presented with patches of hyper- and hypo-reflective cones that qualitatively appeared abnormal (Fig. 3). OCTs from CHM participants with higher ORG amplitudes showed a larger lateral extent of intact retinal lamination in comparison with CHM participants with lower ORG amplitudes which showed the atrophic border encroaching into the central retina.

**Figure 4:**
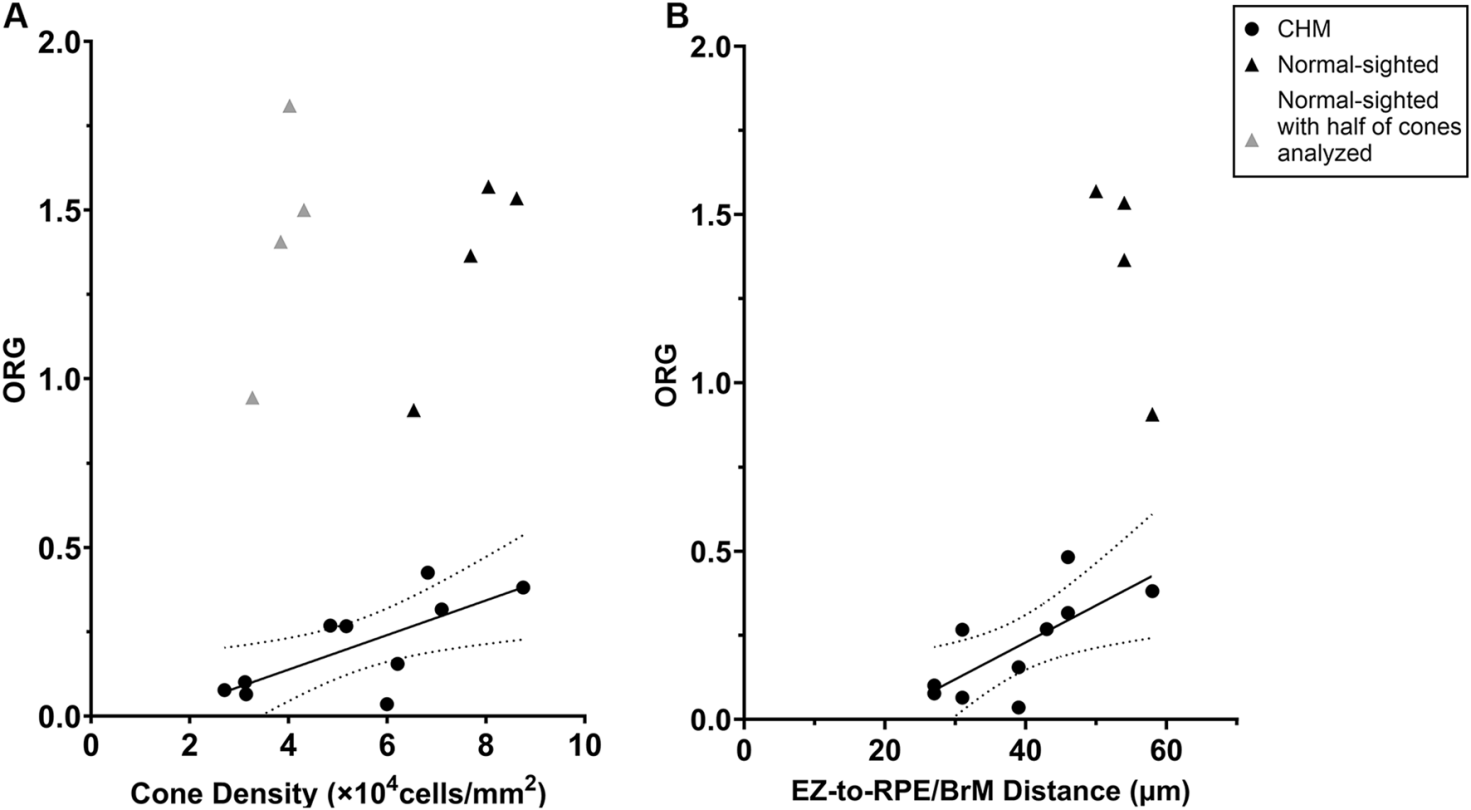
ORG amplitude shows a correlation with cone density (**A**) and EZ-to-RPE/BrM distance (**B**) for CHM participants. The solid line is a linear fit to the CHM data; the dotted curves correspond to the 95% confidence interval of the fit. Pearson correlation coefficient = 0.69 for cone density and 0.72 for EZ-to-RPE/BrM distance. Circles represent data from CHM participants whereas triangles indicate data from normal-sighted participants. Gray triangles represent data from the same normal-sighted participants where only half of the cones were included in the ORG analysis.

To quantitatively assess cone structure in comparison with the ORG, we extracted bound cone densities from regions of interest (ROI) in the AO images and EZ-to-RPE/BrM distances in the OCT images where ORG measurements were acquired. As has been found in other studies, bound cone density was reduced on average in CHM participants compared to normal-sighted participants (CHM: 53,900 ± 19,500 cones/mm^2^, normal-sighted: 77,300 ± 8,800 cones/mm^2^, p = 0.045). Individually, however, CHM participants revealed a range of cone densities with 5 out of 10 CHM participants exhibiting a cone density that fell within the normal range for the parafoveal retinal eccentricities studied (normal density at 150 µm: 87,600 ± 13,400 cones/mm^2^, at 300 µm: 58,400 ± 9,390 cones/mm^2^)^46^. Regardless of cone density, all CHM participants exhibited a reduced ORG amplitude compared to normal-sighted participants. For the CHM participants, cone density showed a moderate positive correlation with ORG amplitude (PCC = 0.69), with higher cone density measurements corresponding to higher ORG amplitudes (Fig. 4A). The EZ-to-RPE/BrM distance was reduced in CHM participants in comparison to normal-sighted participants (CHM: 38.7 ± 9.9, normal-sighted: 54.0 ± 3.3, p = 0.012). For CHM participants, EZ-to-RPE/BrM distance exhibited a high positive correlation with ORG amplitude (PCC = 0.72, Fig. 4B).

**Figure 5:**
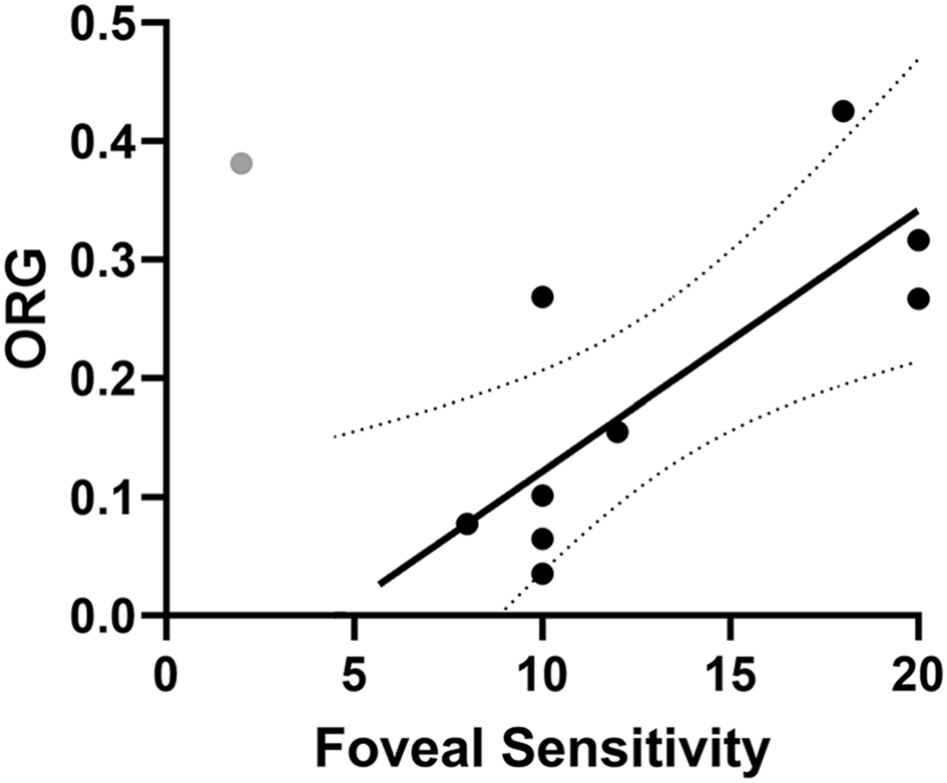
Foveal sensitivity is correlated with ORG amplitude for CHM participants. The solid line is a linear fit and the dotted curves correspond to the 95% confidence interval of the fit. Pearson correlation coefficient = 0.77. The data from participant 13286 (gray circle) was determined to be an outlier by Q test and therefore was excluded from the fit.

Microperimetry revealed constricted visual fields and reduced retinal sensitivities in CHM. Eight of ten CHM participants exhibited a reduced foveal sensitivity as measured on the Nidek MP-1 while two of ten CHM participants demonstrated the normal foveal sensitivity of 20 dB (Table 1). ORG amplitude showed a high positive correlation with foveal sensitivity in the CHM patients (Fig. 5). Linear regression analysis showed foveal sensitivities increased with increasing ORG amplitude (PCC = 0.77). The foveal sensitivity measurement from CHM participant 13286 was classified as an outlier based on Q test with 99% confidence interval and thus was excluded from the linear regression analysis.

## Discussion

Optoretinography has recently emerged as a technique for measuring photoreceptor function in the living human retina^29–35,37,39,47^. In the present study, ORG signals in parafoveal retina were successfully obtained using AOSLO in both normal-sighted participants and those diagnosed with CHM. Though the present study did not investigate the origin of the ORG signal, knowledge of how this signal arises is needed to interpret the current findings. Seminal work from Cooper et al. linked ORG signals with phototransduction, by showing the ORG action spectrum for a population of cones matched the human photopic luminosity function^30^. Since that study, numerous publications have demonstrated the capability of assessing individual photoreceptor function through ORG measurement techniques^29,33,34,37^. Phase based imaging techniques such as AO-OCT and AO line scan ophthalmoscopy have shown that the ORG signal arises from a change in the optical path length (OPL) in nm of the photoreceptor outer segments in response to visible stimulation, and that the cone outer segment OPL is determined by the outer segment refractive index and the geometric distance between the inner segment/outer segment (IS/OS) junction and the cone outer segment tip (COST)^32–35,37^. Recent studies suggest that the measured change in outer segment OPL following light stimulation is a change in the physical length of the outer segment as a result of water diffusing into the outer segment^48–50^, though changes in the refractive index, as may be caused by changes in biomolecules or ion flux with stimulation, have also been suggested^32^. Changes in the OPL of the outer segment can also account for the intensity variations measured in arbitrary units with AOSLO techniques, as cone intensity in AOSLO images is known to be a combination of backscattered light from the cone IS/OS junction and COST layers and the interference between the multiple layers of backscattered light will change as the OPL between the layers changes^31,35^.

For normal-sighted participants, ORG amplitudes were comparable to previously published data using similar stimulus conditions^30^. Previous study has shown ORG amplitudes in normal-sighted participants are repeatable both within and across sessions and that individual variability between subjects is larger than the variability within or across sessions for a single subject^31^. The present study did not test for repeatability, however, multiple stimulation acquisitions were pooled in the analysis to extract the ORG for each participant (Fig. 1D). Inspection of these individual acquisitions (Supplemental Fig. 1) suggest the results of the present study are also repeatable. In the present study, three of the four normal-sighted participants exhibited a larger ORG amplitude than the fourth participant. ORG amplitudes in previous studies likewise have shown similar ranges across individuals, with ORG amplitudes in some cases varying by a factor of two^30,31^. The cause of this inter-subject variability is unknown though possible contributing factors include outer segment length, axial length, and dilated pupil size^39^ in additional to biological variables such as age and sex. In the present study, the normal-sighted participant with the lowest ORG amplitude was female. We decided to include this individual in the dataset, despite the fact that CHM as an X-linked disease affects males, for the following reasons: (1) Removing the female normal-sighted participant from the study would have reduced the normal range of ORG amplitudes reported and as described above ORG amplitudes are known to vary across individuals in similar magnitudes to the present study. (2) Excluding the lowest ORG amplitude from the normal-sighted group would have enhanced the reported difference with the CHM group. (3) Including the female normal-sighted participant did not change the finding that ORG amplitude was reduced in CHM. Future studies to establish ORG norms across these variables will be needed to translate ORG techniques to more widespread clinical use.

We did not assess the within session or across session repeatability in the CHM subjects as was previously done for normal-sighted participants^31^. However, the individual stimulation acquisitions for CHM participants (Supplemental Fig. 1) suggest the ORG is also repeatable in this patient group. We note that in the absence of visible stimuli, cone intensities in the AOSLO video sequences fluctuated more in images from CHM patients than in images from normal-sighted participants (Fig. 2B, t < 0 s). Changes in the measured cone intensities of AOSLO images can result from several factors other than changes in the OPL of the outer segment, including changes in AO wavefront correction, eye movements, and tear film disruptions. As the ORG analysis subtracts the non-stimulated control signal from the stimulated signal to yield the final ORG trace, differences between the CHM and normal-sighted conditions could contribute to part of the difference in the measured ORG amplitudes between the two groups. This effect however, is unable to account for the full difference in ORG amplitude measured between groups as the magnitude of the intensity differences in the unstimulated condition is significantly lower than the difference between the measured ORG amplitudes.

In CHM participants, ORG amplitudes were significantly reduced compared to normal-sighted participants in response to the same visible stimulus. Indeed, ORG amplitudes for CHM subjects were all very low, even in participants who presented in early stages of the disease with high acuities, high sensitivities, and large areas of retained central islands (Table 1, Fig. 3). In this regard, ORG may be useful for monitoring disease state and progression early in the disease process, but may be limited in later stages of the disease when the ORG is essentially flat. We note that using the peak of a gamma-pdf fit as the ORG amplitude, results in a non-zero positive value for all ORG amplitudes. We thus considered ORG amplitudes less than 0.01 to be flat ORGs (Supplemental Fig. 1).

Clinical OCTs in CHM have shown that the interdigitation layer (alternatively named the COST) is the first layer of the OCT to be disrupted within the centrally retained retinal area and that disease severity increases as the retained area decreases^7,12,15^. It has been shown that cones of patients with CHM display different cellular morphological features even within the centrally relatively preserved retinal area. Compared to the cone mosaics of normal-sighted participants, CHM mosaics show reduced cone densities on average, with larger cone inner segments, and altered outer segment waveguiding including patches of hyper- and hypo-reflective cones^9,11^. Such structural features presumably indicate cone degeneration, and may contribute to reduced function. Indeed, the present study demonstrated both a qualitative (Fig. 3) and quantitative (Fig. 4) correlation between ORG amplitude and the severity of the structural phenotype in the CHM parafoveal cone mosaic and the severity of the structural phenotype observed on OCT.

Quantitatively, we found a high positive correlation between the ORG amplitude and the EZ-to-RPE/BrM distance. Clinical OCTs in disease do not always exhibit the retinal layer attributed to the photoreceptor interdigitation zone, especially in CHM as this layer. We thus used the EZ-to-RPE/BrM distance as a quantity that is both measurable in our study participants and is related to, though not precisely, the outer segment length. The EZ-to-RPE/BrM distance was reduced in our CHM participants in comparison to the normal-sighted participants as expected, but not to the same extent as the ORG amplitudes. Future studies using techniques with better axial resolution than our available OCTs, for instance studies using AO-OCT imaging, may provide more accurate and direct measures of outer segment length in CHM. Further study is also needed to understand the impact of outer segment length on ORG amplitude.

We also found a moderate positive correlation between ORG amplitude and cone density for the CHM participants (Fig. 4). On average, cone density was lower in the CHM patients than in the normal-sighted participants. To ensure ORG measurements were not impacted by cone densities in general, we reanalyzed ORG amplitudes using only one-half of the cone identifications acquired from normal-sighted participants. Normal-sighted participant ORG amplitudes analyzed over ½ the densities were not reduced compared to ORG measurements using the full density (P = 0.77; Fig. 4, gray diamonds). Thus, the number of cones used in the ORG measurement is not the cause of the reduced ORG amplitudes for CHM participants. Reduced central cone density has been proposed as a marker of disease severity in CHM^10^. The positive correlation between cone density and ORG amplitude gives credence to the idea that ORG amplitude may also be a marker of disease severity in this patient population. In our CHM cohort, half of the participants in the present study retained normal cone densities. Even for these individuals, the population cone ORG was significantly lower compared to normal-sighted participants. This provides additional evidence for the idea that cone function may be altered in the central retina of CHM patients prior to observable cone loss. Studies which evaluate the ORG in comparison with structural phenotypes of variable severity may help to elucidate the timing between structural and functional degeneration.

Prior studies have established the ORG as a physiological response in stimulated photoreceptors^29,30,34,51^, but it remains to be established whether the ORG is correlated with vision. As a subjective procedure requiring participant input, responses to microperimetry stimuli require perception, and thus threshold sensitivity measurements represent function of the full visual system. In the present study, ORG amplitude showed a high positive correlation with foveal sensitivity (Fig. 5). We use the guidelines proposed by Mukaka to assess the strength of correlations with moderate positive correlations resulting in PCC values from 0.50–0.70 and high positive correlations resulting in PCC values from 0.70–0.90^52^. ORG amplitudes in CHM participants correlated most strongly with foveal sensitivity (0.77) in comparison to the structural measures of cone density (0.69) and EZ-to-RPE/BrM distance (0.72). This finding provides evidence that a reduction in the ORG may represent a surrogate biomarker for a reduction in vision, similar to a reduction in an ERG.

The foveal sensitivity of one CHM patient (13286) in the present study was considered an outlier by Dixon’s Q test and was excluded from the linear regression analysis. On inspection, the microperimetry result acquired from the Nidek of this patient displayed an unusual pattern of sensitivities across the centrally intact retina, with high variability between adjacent test locations. Microperimetry from the Macular Integrity Assessment (MAIA) device acquired from the patient on the same day, however, showed a pattern of sensitivity that systematically decreased from the parafovea to retinal locations further from the fovea, as generally expected for CHM^53^. Thus, we believe the sensitivity threshold measured with the Nidek for this patient to be inaccurate, but have reported its value for completeness in the study. As study procedures generally followed long clinical visits for the CHM patients, we attribute this inaccuracy to patient fatigue. Subjective measurements such as this one strengthen the rationale for an objective measure of visual function.

To our knowledge, there has only been one other report using ORG to assess cone function in retinal disease. Using structural comparisons in three patients with retinitis pigmentosa, Lassoued et al., showed the ORG decreased with increasing disease severity both within and across patients^40^. Similarly, the present study showed the ORG correlated with disease severity using both structural and functional measures in CHM.

The demand for reliable and sensitive outcome measures of visual function remains high. Past clinical trials, such as those testing gene therapies in CHM, have used visual acuity and microperimetry sensitivity as the functional outcome measures for assessing therapeutic efficacy^17,19–21^. The natural history of CHM however, shows these measures remain high until late stages of disease^15,16^. Though much work remains to be done before ORG can be considered an outcome measure for clinical trials, the potential of the ORG as a sensitive biomarker of cone function must be recognized. The present study showed the ORG was reduced even in cases where visual acuity was 20/20 and foveal sensitivity approached normal values. This leads to the idea that the ORG could show improvements following a treatment where other outcome measures may have limited range. Altogether, ORG shows remarkable potential as an objective, sensitive, surrogate measure of photoreceptor function that may become an important addition to the variety of procedures currently used to assess blinding disease and its treatment.

## Conclusions

ORG showed reduced parafoveal cone function in CHM within the centrally intact retinal area, even in patients with normal cone density. Furthermore, ORG amplitude was positively correlated with cone density, EZ-to-RPE/BrM distance, and foveal sensitivity. ORG shows promise as a sensitive, objective biomarker for assessing photoreceptor function in health and disease.

### Supplementary Information

Supplementary Information.

## Data Availability

The datasets generated and analyzed during the current study are available from the corresponding author on reasonable request.

## References

[1] Mura M, Sereda C, Jablonski MM, MacDonald IM, Iannaccone A (2007). Clinical and functional findings in choroideremia due to complete deletion of the CHM gene. Arch. Ophthalmol..

[2] Preising M, Ayuso C (2004). Rab escort protein 1 (REP1) in intracellular traffic: A functional and pathophysiological overview. Ophthalmic Genet..

[3] MacDonald, I. M., Hume, S., Zhai, Y. & Xu, M. Choroideremia. *GeneReviews((R))* (eds M. P. Adam *et al.*) (University of Washington, Seattle, WA, 1993). 20301511

[4] Coussa RG, Traboulsi EI (2012). Choroideremia: A review of general findings and pathogenesis. Ophthalmic Genet..

[5] Heon E (2016). Visual function and central retinal structure in Choroideremia. Investig. Ophthalmol. Vis. Sci..

[6] Jacobson SG (2006). Remodeling of the human retina in choroideremia: rab escort protein 1 (REP-1) mutations. Investig. Ophthalmol. Vis. Sci..

[7] Morgan JI (2014). High-resolution adaptive optics retinal imaging of cellular structure in choroideremia. Investig. Ophthalmol. Vis. Sci..

[8] Morgan JIW, Chui TYP, Grieve K (2023). Twenty-five years of clinical applications using adaptive optics ophthalmoscopy [Invited]. Biomed. Opt. Express.

[9] Xu P, Jiang YY, Morgan JIW (2023). Cone photoreceptor morphology in choroideremia assessed using non-confocal split-detection adaptive optics scanning light ophthalmoscopy. Investig. Ophthalmol. Vis. Sci..

[10] Wynne N, Jiang YY, Aleman TS, Morgan JIW (2023). Foveal Phenotypes in choroideremia on adaptive optics scanning light ophthalmoscopy. Retina.

[11] Sun LW (2016). Multimodal imaging of photoreceptor structure in Choroideremia. PLoS one.

[12] Syed R (2013). High-resolution images of retinal structure in patients with choroideremia. Investig. Ophthalmol. Vis. Sci..

[13] Pennesi ME, Birch DG, Duncan JL, Bennett J, Girach A (2019). CHOROIDEREMIA: Retinal degeneration with an unmet need. Retina.

[14] Coussa RG, Kim J, Traboulsi EI (2012). Choroideremia: effect of age on visual acuity in patients and female carriers. Ophthalmic Genet..

[15] Aleman TS (2017). Natural history of the central structural abnormalities in Choroideremia: A prospective cross-sectional study. Ophthalmology.

[16] Shen LL (2021). Long-term natural history of visual acuity in eyes with choroideremia: A systematic review and meta-analysis of data from 1004 individual eyes. Br. J. Ophthalmol..

[17] MacLaren RE (2014). Retinal gene therapy in patients with choroideremia: Initial findings from a phase 1/2 clinical trial. Lancet.

[18] Xue K (2018). Beneficial effects on vision in patients undergoing retinal gene therapy for choroideremia. Nat. Med..

[19] Aleman TS (2022). Adeno-associated virus serotype 2-hCHM subretinal delivery to the macula in Choroideremia: Two-year interim results of an ongoing phase I/II gene therapy trial. Ophthalmology.

[20] MacLaren RE (2023). Subretinal timrepigene emparvovec in adult men with choroideremia: A randomized phase 3 trial. Nat. Med..

[21] Dimopoulos IS, Tseng C, MacDonald IM (2016). Microperimetry as an outcome measure in Choroideremia trials: Reproducibility and beyond. Investig. Ophthalmol. Vis. Sci..

[22] Jolly JK, Xue K, Edwards TL, Groppe M, MacLaren RE (2017). Characterizing the natural history of visual function in choroideremia using microperimetry and multimodal retinal imaging. Investig. Ophthalmol. Vis Sci.

[23] Tuten WS (2019). Visual function at the atrophic border in choroideremia assessed with adaptive optics Microperimetry. Ophthalmol. Retina.

[24] Hood DC, Birch DG (1990). A quantitative measure of the electrical activity of human rod photoreceptors using electroretinography. Vis. Neurosci..

[25] Creel DJ, Levin KH, Chauvel P (2019). Electroretinograms. Handbook of Clinical Neurology.

[26] Renner AB (2006). Choroideremia: Variability of clinical and electrophysiological characteristics and first report of a negative electroretinogram. Ophthalmology.

[27] Sieving PA, Niffenegger JH, Berson EL (1986). Electroretinographic findings in selected pedigrees with Choroideremia. Am. J. Ophthalmol..

[28] Cheung MC (2004). Detection of localized retinal dysfunction in a choroideremia carrier. Am. J. Ophthalmol..

[29] Cooper RF, Brainard DH, Morgan JIW (2020). Optoretinography of individual human cone photoreceptors. Opt. Express.

[30] Cooper RF, Tuten WS, Dubra A, Brainard DH, Morgan JIW (2017). Non-invasive assessment of human cone photoreceptor function. Biomed. Opt. Express.

[31] Warner RL, Brainard DH, Morgan JIW (2022). Repeatability and reciprocity of the cone optoretinogram. Biomed. Opt. Express.

[32] Pandiyan VP (2020). High-speed adaptive optics line-scan OCT for cellular-resolution optoretinography. Biomed. Opt. Express.

[33] Pandiyan VP (2020). The optoretinogram reveals the primary steps of phototransduction in the living human eye. Sci. Adv..

[34] Zhang F, Kurokawa K, Lassoued A, Crowell JA, Miller DT (2019). Cone photoreceptor classification in the living human eye from photostimulation-induced phase dynamics. Proc. Natl. Acad. Sci. USA.

[35] Jonnal RS (2007). In vivo functional imaging of human cone photoreceptors. Opt Express.

[36] Hillmann D (2016). In vivo optical imaging of physiological responses to photostimulation in human photoreceptors. Proc. Natl. Acad. Sci. USA.

[37] Azimipour M (2020). Optoretinogram: optical measurement of human cone and rod photoreceptor responses to light. Opt. Lett..

[38] Pandiyan VP, Jiang X, Kuchenbecker JA, Sabesan R (2021). Reflective mirror-based line-scan adaptive optics OCT for imaging retinal structure and function. Biomed. Opt. Express.

[39] Jiang X, Liu T, Pandiyan VP, Slezak E, Sabesan R (2022). Coarse-scale optoretinography (CoORG) with extended field-of-view for normative characterization. Biomed. Opt. Express..

[40] Lassoued A (2021). Cone photoreceptor dysfunction in retinitis pigmentosa revealed by optoretinography. Proc. Natl. Acad. Sci. USA.

[41] Dubra A, Sulai Y (2011). Reflective afocal broadband adaptive optics scanning ophthalmoscope. Biomed. Opt. Express..

[42] Scoles D (2014). In vivo imaging of human cone photoreceptor inner segments. Investig. Ophthalmol. Vis. Sci..

[43] Dubra A, Harvey Z (2018). Biomedical Image Registration.

[44] Cunefare D (2017). Open source software for automatic detection of cone photoreceptors in adaptive optics ophthalmoscopy using convolutional neural networks. Sci. Rep..

[45] Tam J, Martin JA, Roorda A (2010). Noninvasive visualization and analysis of parafoveal capillaries in humans. Investig. Ophthalmol. Vis. Sci..

[46] Cooper RF, Wilk MA, Tarima S, Carroll J (2016). Evaluating descriptive metrics of the human cone mosaic. Investig. Ophthalmol. Vis. Sci..

[47] Ma G, Son T, Kim TH, Yao X (2021). In vivo optoretinography of phototransduction activation and energy metabolism in retinal photoreceptors. J. Biophotonics.

[48] Zhang P (2017). In vivo optophysiology reveals that G-protein activation triggers osmotic swelling and increased light scattering of rod photoreceptors. Proc. Natl. Acad. Sci. USA.

[49] Bizheva K (2006). Optophysiology: depth-resolved probing of retinal physiology with functional ultrahigh-resolution optical coherence tomography. Proc. Natl. Acad. Sci. USA.

[50] Yao XC, Yamauchi A, Perry B, George JS (2005). Rapid optical coherence tomography and recording functional scattering changes from activated frog retina. Appl. Opt..

[51] Pandiyan VP (2022). Characterizing cone spectral classification by optoretinography. Biomed. Opt. Express..

[52] Mukaka MM (2012). Statistics corner: A guide to appropriate use of correlation coefficient in medical research. Malawi Med. J..

[53] Poli FE (2023). Correlation between fundus autofluorescence pattern and retinal function on microperimetry in Choroideremia. Trans. Vis. Sci. Technol..

