# Supplementary material for: Parafoveal cone function in choroideremia assessed with adaptive optics optoretinography
Source: Sci Rep. 2024 Apr 9;14:8339. doi: 10.1038/s41598-024-58059-x (PMC11004114; doi:10.1038/s41598-024-58059-x)

**Supplemental Table 1** Number of stimulus and control trials collected, number of acquisitions in each trial and total number of acquisitions analyzed in each CHM participant.

| Subject ID | Number of Stimulus Trials | Number of Acquisitions per Trial | Total Number of Stimulus Acquisitions | Total Number of Control  Acquisitions | Number of Analyzed Stimulus Acquisitions | Number of Analyzed Control Acquisitions |
| --- | --- | --- | --- | --- | --- | --- |
| 13048 | 1 | 10 | 10 | 0 | 7 | 0 |
| 13057 | 3 | 13 | 39 | 26 | 36 | 24 |
| 13125 | 6 | 1 | 6 | 6 | 6 | 6 |
| 13159 | 6 | 1 | 6 | 6 | 6 | 6 |
| 13183 | 2 | 13 | 26 | 13 | 25 | 11 |
| 13195 | 1 | 6 | 6 | 6 | 5 | 5 |
| 13249 | 6 | 1 | 6 | 6 | 5 | 6 |
| 13262 | 4 | 13 | 52 | 13 | 51 | 10 |
| 13278 | 3 | 13 | 39 | 0 | 35 | 0 |
| 13286 | 5 | 13 | 65 | 13 | 54 | 12 |

**Supplemental Figure 1**: Individual stimulation acquisitions (**left**), ORG trace (black line), gamma-pdf fit (green line), and ORG amplitude (red asterisk) (**right**) for each study participant (**A**: normal-sighted, **B**: CHM).


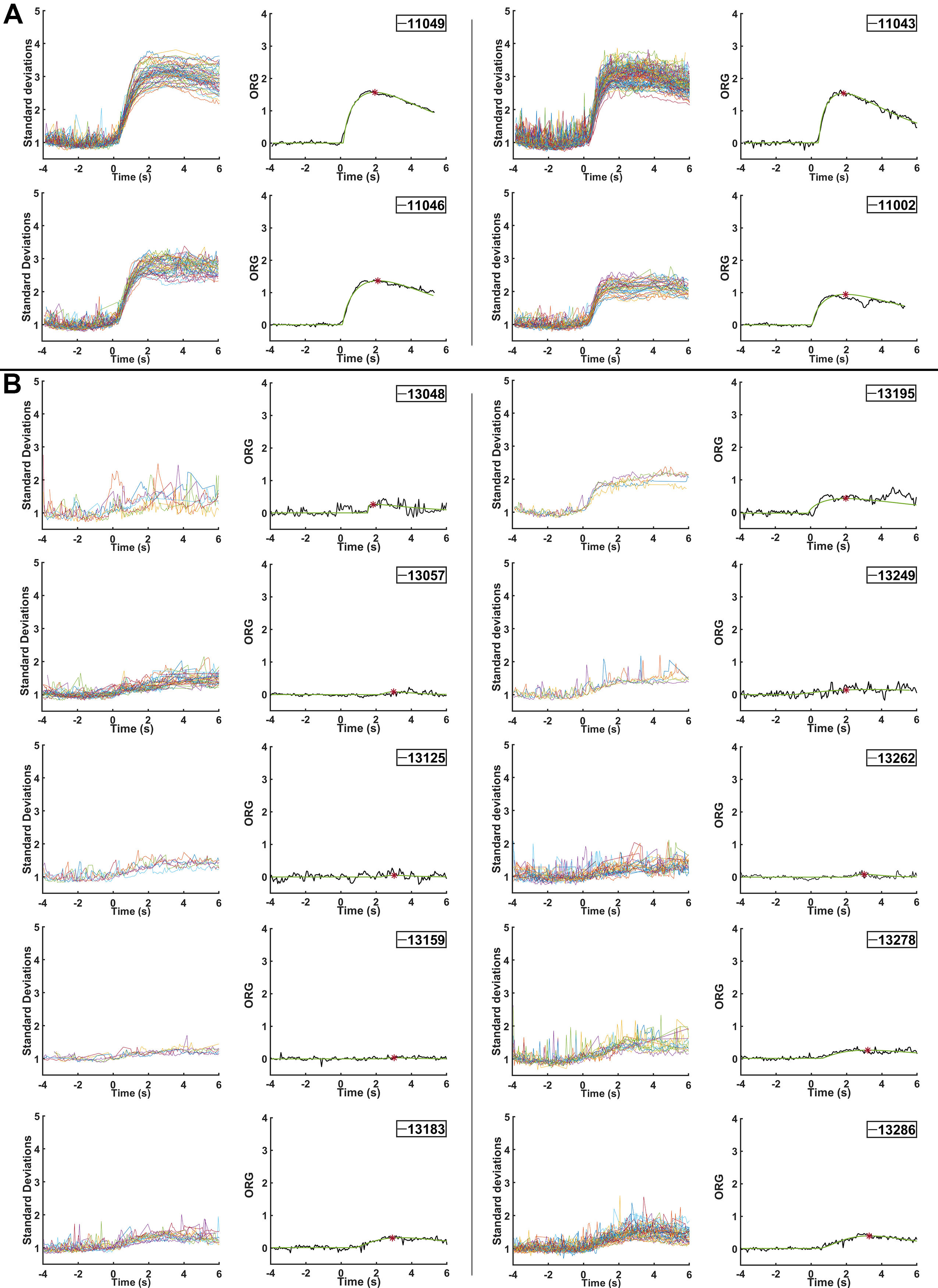

Supplement: Supplementary file 1 — Supplementary Information. [file 41598_2024_58059_MOESM1_ESM.docx]
